# The Relationship Between Physical Activity and Subjective Well‐Being in College Students Over the Course of a Semester: A Short‐Term Longitudinal Study

**DOI:** 10.1002/pchj.70049

**Published:** 2025-08-14

**Authors:** Zhanjia Zhang, Kaijun Wang, Zhonghui He, Xin Qi

**Affiliations:** ^1^ Department of Physical Education Peking University Beijing China; ^2^ School of Leisure Sports Jilin Sport University Changchun China

**Keywords:** college students, physical activity, subjective well‐being

## Abstract

Subjective well‐being is an essential construct in positive psychology and is linked with various physical and mental health outcomes in college students. This study examined the associations between different intensities of physical activity (PA) and multiple dimensions of subjective well‐being at the beginning and end of a semester. A short‐term longitudinal design was employed with a cohort of 743 undergraduate students (mean age = 19.63 years). Data on PA and subjective well‐being indicators, including life satisfaction, positive affect, and negative affect, were collected during the third week of the semester and the week before final exams. The results revealed a significant decline in PA and life satisfaction, and a significant increase in negative affect from the beginning to the end of the semester. Vigorous‐intensity PA at the semester's end showed a positive relationship with life satisfaction and positive affect, and a negative relationship with negative affect, while PA levels at the beginning of the semester did not predict subjective well‐being at the semester's end. This study highlights the importance of vigorous‐intensity PA in supporting subjective well‐being during periods of academic stress. Universities should implement targeted programs to encourage vigorous‐intensity PA to support student well‐being.

## 1 Introduction

College students experience a pivotal growth period as they transition from late adolescence to the early stages of adulthood. This stage of emerging adulthood is often accompanied by various challenges, such as navigating personal identity, building independence, and managing new social relationships (Arnett et al. 2014). College life itself can be particularly demanding, as students face academic pressures, social integration difficulties, and concerns about their future careers (Hurst et al. 2013). These numerous sources of stress can lead to significant mental health issues among college students, such as depression and anxiety. According to a nationwide survey, 60% of students reported experiencing at least one mental health problem, with 36% and 41% of them reporting anxiety and depressive symptoms, respectively (Lipson et al. 2022). The rising prevalence of mental health issues among college students has drawn the attention of academic institutions, mental health professionals, and the broader public. Numerous initiatives have been implemented to address these concerns by providing resources, support systems, and awareness campaigns to improve mental well‐being on campuses (Cuijpers et al. 2019).

Previous studies concerning the mental health of college students have largely centered around negative psychological states like depression and anxiety, neglecting the positive aspects of mental health (Carver et al. 2021). This approach has led to the assumption that students are mentally healthy as long as they do not exhibit negative psychological symptoms. In recent years, as the importance of positive psychology has been increasingly recognized, there has been a growing interest in exploring positive mental well‐being among college students (Flett et al. 2019). A central concept within positive psychology is subjective well‐being, which is linked to a wide range of favorable physical and mental health outcomes (Das et al. 2020).

Subjective well‐being refers to an individual's general appraisal of their life, including both cognitive and affective components (Diener 1984). The cognitive component, often termed as life satisfaction, involves a reflective judgment about one's overall life quality based on personal standards and expectations. It provides an overarching view of how satisfied individuals are with their lives as a whole. On the other hand, the affective component involves the emotional aspects of well‐being, which include both positive affect (emotions and moods such as joy, contentment, and enthusiasm) and negative affect (experiences of distress and discomfort, such as sadness, anger, and anxiety). Both components are crucial in understanding college students' comprehensive mental well‐being and are linked to numerous positive outcomes, such as academic performance and social integration (Bücker et al. 2018; Imaginário et al. 2013).

Researchers have long been interested in the factors that influence subjective well‐being. Physical activity (PA), defined as “any bodily movement produced by skeletal muscles that requires energy expenditure (Caspersen et al. 1985)”, is one of the key factors that have been shown to positively affect subjective well‐being (Buecker et al. 2021). Despite the well‐known health benefits of PA, a large proportion of college students fail to achieve the recommended weekly level of 150 min of moderate‐to‐vigorous intensity PA (Bailey et al. 2022). There has been a growing body of research investigating the effects of PA on college students' subjective well‐being. For example, Piqueras et al. (2011), in their cross‐sectional research, discovered that Chilean college students engaging in daily PA had a 1.3 times higher likelihood of reporting happiness than those who did not. Grant et al. (2009) examined college students from 21 countries and found that those who did not engage in sufficient leisure‐time PA exhibited significantly reduced life satisfaction compared to their peers who were more physically active.

Despite the growing interest in the association between PA and subjective well‐being among college students, much of the existing research has been limited in scope, often examining only one component of subjective well‐being. Furthermore, many studies have relied on cross‐sectional designs, focusing on the relationship between PA and subjective well‐being at a single time point, without accounting for how this relationship may evolve over the course of a semester. Given that students may experience varying levels of stress due to multiple factors—such as academic demands, financial concerns, and social transitions—examining PA and well‐being across different time points can offer a more nuanced understanding of these associations. Previous literature has pointed to potential increases in stress toward the end of a semester (Baghurst and Kelley 2014; Pitt et al. 2018). The heightened stress during the end of a semester can lead to several adverse outcomes, such as increased substance use and suicidal ideation (Khalifah et al. 2023; Yaylaci 2015). The cross‐stressor adaptation hypothesis (Sothmann et al. 1996) suggests that the physiological adaptations from regular PA may enhance an individual's ability to respond to various stressors; but this adaptation effect may be particularly relevant during periods of heightened challenge. Excessive stress is also known to negatively impact subjective well‐being in college students (Lane et al. 2020), emphasizing the need to better understand how subjective well‐being is affected during high‐stress periods.

Understanding how both PA and subjective well‐being change and interact throughout a college semester is crucial for developing effective interventions. Research suggests that college students' PA levels typically decline over the course of a semester (Harari et al. 2017). Similarly, subjective well‐being tends to fluctuate throughout the semester, with studies documenting decreases in subjective well‐being as the semester progresses (De Coninck et al. 2019). Thus, examining how these associations differ across time points may help to clarify the temporal pattern of the relationship and identify periods where PA could be particularly beneficial for student well‐being.

PA is a complex behavior, and its intensity represents an important dimension that may influence its relationship with subjective well‐being. PA intensity is typically categorized into light, moderate, and vigorous based on metabolic equivalent values and perceived exertion (Norton et al. 2010). Research suggests that PA of different intensities may have distinct psychological effects. For example, Zhang et al. (2022) found that moderate‐to‐vigorous intensity PA was more strongly associated with subjective well‐being than light‐intensity PA, while Wicker and Frick (2017) observed that light‐intensity PA and moderate‐intensity PA had positive associations with subjective well‐being. These mixed findings highlight the importance of considering intensity when examining the relationship between PA and subjective well‐being.

Taken together, exploring the relationship between PA and subjective well‐being at different times within a semester is an important issue that warrants the attention of researchers and related professionals. Given the limitations of previous studies, the current study aimed to assess college students' PA and subjective well‐being at the beginning of the semester and again 1 week before final exams. These specific points were selected because the end of the semester is often reported by students as the most stressful period (Baghurst and Kelley 2014; Beiter et al. 2015), allowing us to investigate how PA might influence subjective well‐being over time.

Building on previous evidence, we developed several hypotheses for this study. First, we hypothesized that PA would decrease in the week before final exams compared to the beginning of the semester. Second, we expected that subjective well‐being would also decline, as reflected by reduced life satisfaction and positive affect, along with increased negative affect. Third, we expected that PA would show a cross‐sectional association with subjective well‐being at both the semester's start and end. Finally, we hypothesized that even after controlling for initial levels of PA and subjective well‐being, PA at the end of the semester would still significantly predict higher levels of subjective well‐being. However, the specific relationship between the PA intensity and different components of subjective well‐being remains uncertain, as previous studies have reported mixed results (Wiese et al. 2018). The findings of our study could help inform the development of effective strategies to enhance students' well‐being through appropriately tailored PA programs.

## 2 Methods

### 2.1 Study Design and Participants

This study employed a short‐term longitudinal design, with data collected at two time points within a fall semester in Beijing, a northern Chinese city. Students from a large university were recruited through a required course offered to all students. Researchers visited the classroom to explain the study's purpose and procedures in detail. It was emphasized that their participation in the study was completely voluntary and would have no impact on their course grades. Participants were also assured that their confidentiality would be strictly maintained. The initial data collection (Time 1) occurred in the third week of the semester, during which participants completed questionnaires evaluating their PA and subjective well‐being. The second data collection (Time 2) occurred during the fifteenth week of the same semester (i.e., the week before final exams), when participants were contacted again to complete the same set of questionnaires. Written informed consent was obtained from all participants before the Time 1 data collection. The study protocol received approval from the institutional review board affiliated with the authors.

A total of 743 students completed both Time 1 and Time 2 assessments and were included in the study's final sample. Among them, there were 378 male students, with a mean age of 19.80 years (SD = 1.36), and 365 female students, with a mean age of 19.50 years (SD = 1.21).

### 2.2 Measures

#### 2.2.1 Physical Activity

PA was assessed with the short form of the International Physical Activity Questionnaire (IPAQ‐SF; Craig et al. 2003). The IPAQ‐SF is a brief, self‐report instrument designed to assess the frequency and duration of PA across different intensities—vigorous, moderate, and light (reflected by walking)—over the past 7 days. Participants were instructed to indicate both the number of days and the average daily time they engaged in each activity. Total minutes per week that participants spent on each intensity of PA were calculated to represent their weekly levels of PA at various intensities. The IPAQ‐SF is one of the most widely used self‐report tools for assessing PA, and its validity for use among Chinese college students has been well established in previous research (Macfarlane et al. 2007).

#### 2.2.2 Life Satisfaction

Life satisfaction was assessed with the Satisfaction with Life Scale (SWLS), a widely recognized self‐report questionnaire designed to evaluate a participant's overall cognitive judgment of their life satisfaction (Diener et al. 1985). Participants respond to five statements, such as “In most ways, my life is close to my ideal,” using a 7‐point Likert scale ranging from 1 (*strongly disagree*) to 7 (*strongly agree*). The overall score is calculated by summing responses to the five items, with higher scores reflecting greater life satisfaction. Previous research has established the SWLS as a reliable and valid measure for Chinese college students (Sachs 2004). In this study, the SWLS demonstrated good internal consistency, with a Cronbach's alpha of 0.86 at Time 1 and 0.89 at Time 2.

#### 2.2.3 Positive Affect and Negative Affect

The Positive and Negative Affect Schedule (PANAS; Watson et al. 1988) was utilized to assess the positive affect and negative affect in participants. The PANAS is a 20‐item self‐report scale consisting of two subscales, each containing 10 items measuring positive affect and negative affect, respectively. It captures a range of affective states that participants have experienced, such as “interested” for positive affect and “distressed” for negative affect. Respondents report their feelings over the past week on a 5‐point Likert scale, from 1 (*very slightly or not at all*) to 5 (*extremely*). Each subscale score is calculated by summing the responses for the relevant items, with higher scores indicating greater levels of positive or negative affect. The PANAS has been validated as an effective tool for assessing the positive affect and negative affect among Chinese college students (Huang et al. 2003). The reliability of PANAS in the current study was satisfactory, with Cronbach's alpha of 0.86 at Time 1 and 0.88 at Time 2 for the positive affect subscale and 0.85 at Time 1 and 0.88 at Time 2 for the negative affect subscale.

#### 2.2.4 Covariates

To account for potential confounding factors, gender and age were included as covariates in the current study, as previous studies have suggested that both gender and age are associated with PA and subjective well‐being (Matud et al. 2022; Trost et al. 2002). Both covariates were self‐reported by participants at the initial data collection.

### 2.3 Statistical Analysis

Descriptive statistics were calculated to provide an overview of the main characteristics of the study variables. The normality of the continuous variables was assessed using skewness and kurtosis values. Skewness between −7 and +7 and kurtosis between −2 and +2 were considered indicative of no significant violation of normality. Paired samples *t*‐tests were conducted to examine changes in PA, life satisfaction, positive affect, and negative affect from the beginning to the end of the semester. Pearson correlation coefficients were computed to assess the bivariate associations among the continuous variables.

To examine the relationship between PA and subjective well‐being at the beginning of the semester, we conducted three multiple linear regression analyses, with life satisfaction, positive affect, and negative affect as the dependent variables in each model, respectively. In all three models, vigorous‐intensity PA, moderate‐intensity PA, and light‐intensity PA were included as predictor variables, while controlling for age and gender to account for their potential confounding effects. To examine the impact of end‐of‐semester PA on subjective well‐being, each end‐of‐semester subjective well‐being indicator was regressed on the end‐of‐semester vigorous‐intensity PA, moderate‐intensity PA, and light‐intensity PA, while controlling for gender, age, the corresponding subjective well‐being indicators at Time 1, and PA of different intensities at Time 1. One potential concern of simultaneously including PA of different intensities and time points in the same model is multicollinearity. We used the Variance Inflation Factor (VIF) to assess multicollinearity in all linear regression models, with VIF values below 5 indicating no severe multicollinearity. All statistical analyses were conducted using R version 4.3, and a significance level of *p* < 0.05 was set for all analyses.

## 3 Results

Table 1 provides descriptive statistics for each study variable at both Time 1 and Time 2, as well as the paired samples t‐test findings. The skewness and kurtosis values for all continuous variables fell within the acceptable ranges, indicating no significant violation of normality. At the beginning of the semester (Time 1), participants reported a mean of 90.64 min per week for vigorous‐intensity PA, 135.84 min per week for moderate‐intensity PA, and 335.67 min per week for light‐intensity PA. For subjective well‐being indicators at Time 1, participants reported mean scores of 22.41 for life satisfaction, 31.72 for positive affect, and 21.25 for negative affect, which were slightly above the midpoints of the SWLS and the Positive Affect subscale of the PANAS, and slightly below the midpoint of the Negative Affect subscale of the PANAS, suggesting that participants generally experienced a moderate‐to‐high level of life satisfaction and positive affect, while experiencing a relatively lower level of negative affect at the beginning of the semester.

**TABLE 1:** Summary of descriptive statistics (mean with standard deviation or counts with percentage) and paired samples t‐test.

Variables	Time 1	Time 2	*t* (df = 742)	*p*	Cohen's *d*
Vigorous‐intensity PA (min/week)	90.64 ± 105.46	61.58 ± 90.46	−7.793	< 0.001	−0.29
Moderate‐intensity PA (min/week)	135.84 ± 124.48	110.05 ± 121.4	−4.922	< 0.001	−0.18
Light‐intensity PA (min/week)	335.67 ± 245.66	267.56 ± 200	−7.675	< 0.001	−0.28
Life satisfaction	22.41 ± 6.1	22.02 ± 6.58	−2.269	0.024	−0.08
Positive affect	31.72 ± 6.2	31.61 ± 6.65	−0.563	0.574	−0.02
Negative affect	21.25 ± 6.12	22.61 ± 6.84	6.483	< 0.001	0.24
Age (years)	19.63 ± 1.3				
Gender					
Male	378	50.9%			
Female	365	49.1%			

As expected, paired samples t‐tests revealed a significant decrease in participants' engagement in vigorous‐intensity PA (90.64 vs. 61.58 min/week, *p* < 0.001), moderate‐intensity PA (135.84 vs. 110.05 min/week, *p* < 0.001), and light‐intensity PA (335.67 vs. 267.56 min/week, *p* < 0.001) from the beginning to the end of the semester. Furthermore, compared to the beginning of the semester, students reported significantly lower life satisfaction (22.41 vs. 22.02, *p* = 0.024) and higher levels of negative affect (21.25 vs. 22.61, *p* < 0.001) at the end of the semester, while no significant change was observed in positive affect (31.72 vs. 31.61, *p* = 0.574). Effect sizes for these comparisons are also reported in Table 1. It should be noted that effect sizes for significant changes were generally small (Cohen's d ranging from 0.18 to 0.29), indicating modest changes over time.

Table 2 presents the bivariate correlations among different intensities of PA and subjective well‐being indicators. The results indicate that not all intensities of PA were significantly associated with all subjective well‐being indicators. Overall, vigorous‐intensity PA demonstrated the strongest correlations with subjective well‐being measures, while light‐intensity PA showed the weakest associations with these indicators. Notably, negative affect was not significantly associated with any intensity of PA at either time point.

**TABLE 2:** Bivariate correlations among PA of different intensities and subjective well‐being indicators.

Variables	1	2	3	4	5	6	7	8	9	10	11	12
1. Vigorous—intensity PA at T1	—											
2. Moderate‐intensity PA at T1	0.24***	—										
3. Light‐intensity PA at T1	0.07	0.16***	—									
4. Life satisfaction at T1	0.07*	0.04	0.03	—								
5. Positive affect at T1	0.18***	0.18***	0.12***	0.51***	—							
6. Negative affect at T1	0.02	−0.01	0.03	−0.37***	−0.03	—						
7. Vigorous‐intensity PA at T2	0.47***	0.16***	0.03	0.06	0.22***	0.04	—					
8. Moderate‐intensity PA at T2	0.24***	0.33***	0.08*	0.13***	0.22***	0.02	0.32***	—				
9. Light‐intensity PA at T2	0.05	0.04	0.43***	0.01	0.05	0.01	0.06	0.15***	—			
10. Life satisfaction at T2	0.11**	0.08*	0.02	0.73***	0.45***	−0.36***	0.14***	0.17***	0.06	—		
11. Positive affect at T2	0.18***	0.15***	0.06	0.48***	0.67***	−0.13***	0.25***	0.26***	0.06	0.62***	—	
12. Negative affect at T2	0.01	0.01	0.01	−0.36***	−0.11**	0.62***	−0.03	−0.04	−0.05	−0.44***	−0.21***	—

*Note*: T1, time 1, beginning of the semester; T2, time 2, end of the semester.

* *p* < 0.05.

** *p* < 0.01.

*** *p* < 0.001.

Table 3 presents the associations between different PA intensities and subjective well‐being indicators at the beginning of the semester. The results indicated that none of the PA intensities were significant predictors of life satisfaction or negative affect (*p*s > 0.05). In contrast, vigorous‐intensity PA (*β* = 0.141, *p* < 0.001), moderate‐intensity PA (*β* = 0.124, *p* < 0.001), and light‐intensity PA (*β* = 0.092, *p* = 0.011) were all found to be significantly and positively correlated with positive affect, indicating that higher engagement in any intensity of PA was related to greater positive affect among participants. Additionally, female participants reported lower levels of positive affect compared to male participants (*β* = −0.145, *p* = 0.044), and older participants tended to have higher levels of negative affect (*β* = 0.076, *p* = 0.042).

**TABLE 3:** Associations (standardized coefficients with standard errors) between PA and subjective well‐being indicators at Time 1.

	Life satisfaction at T1		Positive affect at T1		Negative affect at T1	
	*β*	SE	*β*	SE	*β*	SE
Vigorous‐intensity PA at T1	0.067	0.038	0.141***	0.037	0.023	0.038
Moderate‐intensity PA at T1	0.019	0.039	0.124***	0.037	−0.014	0.039
Light‐intensity PA at T1	0.025	0.037	0.092*	0.036	0.028	0.037
Age	0.003	0.037	−0.068	0.036	0.076*	0.037
Gender	0.028	0.074	−0.145*	0.072	−0.048	0.074
*R*‐squared	0.65%		7.13%		0.81%	
*F* (df_1_ = 5, df_2_ = 737)	0.96		11.23***		1.21	
*N*	743		743		743	

*Note*: T1, time 1, beginning of the semester.

* *p* < 0.05.

*** *p* < 0.001.

The standardized coefficients reflecting the relationships between end‐of‐semester PA at different intensities and subjective well‐being indicators are summarized in Table 4. Notably, vigorous‐intensity PA at Time 2 emerged as a significant predictor of end‐of‐semester life satisfaction (*β* = 0.082, *p* = 0.005), positive affect (*β* = 0.084, *p* = 0.007), and negative affect (*β* = −0.07, *p* = 0.041), even after adjusting for baseline levels of the corresponding subjective well‐being indicators and different intensities of PA at the beginning of the semester. In contrast, moderate‐intensity PA at Time 2 was significantly positively associated only with positive affect at Time 2 (*β* = 0.091, *p* = 0.002). Light‐intensity PA at Time 2, however, showed no significant relationship with any of the subjective well‐being indicators. Additionally, each baseline measure of subjective well‐being was found to significantly predict its corresponding end‐of‐semester indicator, while no PA intensity at the beginning of the semester significantly predicted any of the subjective well‐being indicators at the end of the semester. No multicollinearity issues were detected across all multiple linear regression models, as all VIF values were close to 1.

**TABLE 4:** Associations (standardized coefficients with standard errors) between PA and subjective well‐being indicators at Time 2.

	Life satisfaction at T2		Positive affect at T2		Negative affect at T2	
	*β*	SE	*β*	SE	*β*	SE
Life satisfaction at T1	0.717***	0.025				
Positive affect at T1			0.643***	0.028		
Negative affect at T1					0.621***	0.029
Vigorous‐intensity PA at T1	0.006	0.029	0.000	0.031	0.035	0.034
Moderate‐intensity PA at T1	0.032	0.027	−0.002	0.029	−0.006	0.032
Light‐intensity PA at T1	−0.036	0.028	−0.038	0.03	0.005	0.033
Vigorous‐intensity PA at T2	0.082**	0.029	0.084**	0.032	−0.07*	0.034
Moderate‐intensity PA at T2	0.038	0.028	0.091**	0.03	−0.009	0.033
Light‐intensity PA at T2	0.049	0.028	0.026	0.03	−0.051	0.033
Age	0.031	0.025	0.046	0.027	−0.006	0.03
Gender	−0.009	0.051	0.051	0.055	−0.006	0.059
*R*‐squared	54.55%		47.51%		38.98%	
*F* (df_1_ = 9, df_2_ = 733)	97.75***		73.71***		51.11***	
N	743		743			

*Note*: T1, time 1, beginning of the semester; T2, time 2, end of the semester.

* *p* < 0.05.

** *p* < 0.01.

*** *p* < 0.001.

## 4 Discussion

Although past research has examined the relationship between PA and subjective well‐being in college students, few studies have examined these variables at multiple time points throughout a semester. This study investigated the relationship between different intensities of PA and subjective well‐being among college students over a semester. Overall, our findings partially support our hypotheses. The results showed that vigorous‐intensity, moderate‐intensity, and light‐intensity PA all significantly decreased from the beginning to the end of the semester. This decline in PA levels aligns with prior studies indicating that as the semester progresses, students have less time and motivation to engage in PA (Wunsch et al. 2017).

As the semester progressed, life satisfaction significantly decreased and negative affect significantly increased. While we did not directly measure sources of stress, these patterns are consistent with prior research showing that students' well‐being can decline over the course of a semester (Pitt et al. 2018). Unexpectedly, positive affect did not show a significant change over time. This stability in positive emotions might suggest that positive affect is less sensitive to short‐term stressors or that students have developed adaptive strategies to maintain their positive emotional states. This finding further supports the idea that positive affect and negative affect are independent dimensions of subjective well‐being, rather than opposite ends of a single spectrum (Diener and Emmons 1984).

Our findings show that the relationship between PA and subjective well‐being differs across different time points within the semester. At the beginning of the semester, PA was only associated with positive affect, while at the end of the semester, PA was significantly related to life satisfaction, positive affect, and negative affect. This difference may be due to the varying contexts experienced by students at different points in the semester; though the specific factors driving this change were not directly measured. These findings indicate that measuring PA and subjective well‐being at only a single time point may not provide a comprehensive understanding of their relationship.

An important finding of this study is that the intensity of PA matters for its association with subjective well‐being at the end of the semester. Engaging in vigorous‐intensity PA was a consistent predictor of greater life satisfaction, elevated positive affect, and decreased negative affect. This aligns with previous research showing that vigorous‐intensity PA is associated with increased life satisfaction among adolescents aged 13 to 18 (Shennar‐Golan and Walter 2018). Additionally, vigorous‐intensity PA has also been found to provide other psychological benefits in college students, such as stress reduction (VanKim and Nelson 2013). However, Wicker and Frick (2017) found that moderate‐intensity PA was positively related to subjective well‐being, whereas vigorous‐intensity PA was negatively associated with subjective well‐being among adults with an average age of 51. One possible explanation for these inconsistent findings is the age difference between study populations. Arguably, age‐related factors, such as changes in physical fitness, might make vigorous‐intensity PA feel more strenuous or less enjoyable for older adults, thereby reducing their psychological benefits, while younger individuals, such as college students, may find vigorous PA more energizing and rewarding.

Additionally, although we found that vigorous‐intensity PA at the end of the semester was positively associated with subjective well‐being, none of the PA intensities at the beginning of the semester significantly predicted any of the end‐of‐semester subjective well‐being indicators. This suggests that the benefits of PA for students' subjective well‐being may be more immediate rather than cumulative over time. This finding emphasizes the importance of encouraging participation in PA, particularly during critical high‐stress periods. It should also be noted that PA explained only a small portion of the variance in subjective well‐being indicators in this study. This is reasonable given that subjective well‐being is influenced by a multitude of factors (Diener et al. 2018). Despite the small estimate of the association between PA and subjective well‐being, we still believe this finding could be meaningful, particularly because our study controlled for earlier measures of subjective well‐being. Furthermore, considering the significant role that subjective well‐being plays in predicting critical health outcomes like morbidity and mortality (Martín‐María et al. 2017), even a small enhancement in subjective well‐being through PA could be beneficial for overall health.

The findings of the current study provide potential implications for universities regarding well‐being programs; though causal interpretations must be approached with caution given the observational nature of our study. Given that our data showed associations between vigorous‐intensity PA and various aspects of subjective well‐being, universities might consider exploring programs and initiatives that encourage more vigorous forms of exercise. Furthermore, even during high‐stress periods like final exams, PA engagement might benefit students' subjective well‐being, as suggested by Koschel et al. (2017) who found that students participating in a university PA program during the final exam period reported perceived stress reduction, supporting the potential value of such initiatives.

### 4.1 Limitations and Future Directions

Several limitations of the current study should be noted. First, this study was conducted over a single semester, and the sample was limited to one university in China, which restricts the generalizability of the results. It would be interesting for future studies to explore whether the observed patterns of change in PA and subjective well‐being, as well as their relationships at the end of the semester, can be replicated among college students in different universities or cultural contexts. In addition, our study was conducted during the fall semester in Beijing, where weather conditions change greatly from early semester (September) to the end of semester (December), which may have contributed to reduced PA and lower well‐being over time. Future research could conduct similar studies across different semesters or control for temperature variations to better understand the patterns of PA and subjective well‐being changes across an academic term. Second, the use of self‐reported measures for PA may have introduced recall or social desirability biases, affecting the accuracy of the data. Future research should incorporate objective measures of PA, such as accelerometers or wearable activity trackers, to provide more precise assessments and help validate the findings. Third, our study only measured PA and subjective well‐being at two time points—at the beginning and the end of the semester—which limits our understanding of how these variables fluctuate over shorter periods. Future research could include more frequent measurements, such as week‐to‐week or day‐to‐day assessment, to capture more subtle changes and provide a deeper insight into the temporal relationships between PA and subjective well‐being. Fourth, while we controlled for age and gender, we did not account for other potential confounding variables such as pre‐existing mental health conditions, sleep quality, social support, and socioeconomic status. These unaccounted factors should be considered in future research designs. Future studies should also measure variables that may impact this relationship, such as sources of stress, time availability, and non‐academic life stressors to better isolate the relationship between PA and subjective well‐being.

With these limitations in mind, our study also offers several contributions. First, we employed a longitudinal research design, measuring PA and subjective well‐being at two different time points within the semester, with a focus on the end of the semester. This allows us to capture the changes in PA and subjective well‐being across different periods, providing a more comprehensive understanding of how these variables are associated under varying levels of academic stress. Second, we took into account different intensities of PA and various dimensions of subjective well‐being. Given that PA is a complex behavior involving activities of varying intensity (Rhodes and Nigg 2011), this enabled us to provide a more detailed understanding of which forms of PA are most beneficial for subjective well‐being. Furthermore, by dividing subjective well‐being into its cognitive and affective components, the study offers a more granular perspective on how different aspects of well‐being may be independently influenced by PA.

## 5 Conclusion

The current study is one of the few studies that investigates the relationship between PA and subjective well‐being among college students at multiple points within a semester. We investigated PA and subjective well‐being at the beginning of the semester and again during the week before final exams. The results showed that in a cohort of students, there was a decrease in both PA and subjective well‐being from the beginning to the end of the semester. Vigorous‐intensity PA proved to be most effective in enhancing various aspects of well‐being, such as life satisfaction, positive affect, and reducing negative affect at the end of the semester. University administrators should consider implementing programs and initiatives that promote vigorous‐intensity PA during stressful periods. Such efforts could not only promote a more active lifestyle and enhanced engagement in PA among students but also serve as an effective tool for enhancing their mental well‐being.

## Ethics Statement

All procedures performed in studies involving human participants were in accordance with the ethical standards of the institutional and/or national research committee and with the 1964 Helsinki declaration and its later amendments or comparable ethical standards. Written informed consent was obtained from all participants. The study protocol was approved by the Peking University Institutional Review Board (#20240408).

## Conflicts of Interest

The authors declare no conflicts of interest.

## Data Availability

The dataset analyzed during the current study can be retrieved from the public data repository OSF (https://osf.io/u5qdk/).
